# The Predictivity of N-Terminal Pro b-Type Natriuretic Peptide for All-Cause Mortality in Various Follow-Up Periods among Heart Failure Patients

**DOI:** 10.3390/jcm8030357

**Published:** 2019-03-13

**Authors:** Min-Yu Lai, Wei-Chih Kan, Ya-Ting Huang, John Chen, Chih-Chung Shiao

**Affiliations:** 1 Department of Nursing, Saint Mary’s Hospital Luodong, Luodong, Yilan 26546, Taiwan; smh01603@smh.org.tw (M.-Y.L.); frankie7451@gmail.com (Y.-T.H.); 2 Department of Nephrology, Department of Internal medicine, Chi Mei Medical Center, Yongkang District, Tainan City 710, Taiwan; rockiekan@ntu.edu.tw; 3 Department of Biological Science and Technology, Chung Hwa University of Medical Technology, Rende District, Tainan City 717, Taiwan; 4 Division of Cardiovascular Medicine, Department of Internal Medicine, Saint Mary’s Hospital Luodong, Luodong, Yilan 26546, Taiwan; aqueocean@gmail.com; 5 Division of Nephrology, Department of Internal Medicine, Saint Mary’s Hospital Luodong, Luodong, Yilan 26546, Taiwan; 6 Saint Mary’s Junior College of Medicine, Nursing and Management, Sanxing Township, Yilan County 266, Taiwan

**Keywords:** all-cause mortality, follow-up period, heart failure, hospitalization, NT-proBNP

## Abstract

Plasma N-terminal pro-brain natriuretic peptide (NT-proBNP) is an excellent prognostic–predictive tool in heart failure (HF) patients, but its plasma level changes following therapy. The comparison of prognosis–predictivity of a single measurement of plasma NT-pro BNP in different follow-up periods in acute HF patients has been less studied. This study aimed to evaluate whether the association between initial plasma NT-proBNP levels and all-cause mortality would decrease along with an increased follow-up period in patients with acute HF. The retrospective study was carried out, enrolling adult patients with hospitalization-requiring acute HF who fulfilled the predefined criteria from January 1, 2011, to December 31, 2013. We evaluated the independent predictors of 12-month mortality, and subsequently compared the predictivity of NT-proBNP level at initial presentation for 1-, 3-, 6-, 9- and 12-month mortality. In total, 269 patients (mean age, 74.45 ± 13.59 years; female, 53.9%) were enrolled. The independent predictors of 12-month mortality included higher “Charlson Comorbidity Index” (adjusted hazard ratio (aHR) = 1.22; 95% confidence interval (CI), 1.10–1.34), increased “age” (aHR = 1.07; 95% CI, 1.04–1.10), “administration of vasopressor” (aHR = 3.43; 95% CI, 1.76–6.71), “underwent cardiopulmonary resuscitation” (aHR = 4.59; 95% CI, 1.76–6.71), and without “angiotensin-converting enzyme inhibitors/angiotensin receptor blocker” (aHR = 0.41; 95% CI, 1.86–11.31) (all *p* <0.001). “Plasma NT-pro BNP level ≧11,755 ng/L” was demonstrated as an independent predictor in 1-month (aHR = 2.37; 95% CI, 1.10–5.11; *p* = 0.028) and 3-month mortality (aHR = 1.98; 95% CI, 1.02–3.86; *p* = 0.045) but not in more extended follow-up. The outcome predictivity of plasma NT-proBNP levels diminished in a longer follow-up period in hospitalized acute HF patients. In conclusion, these findings remind physicians to act with caution when using a single plasma level of NT-proBNP to predict patient outcomes with a longer follow-up period.

## 1. Introduction

Heart failure (HF) is a complex and fatal medical entity with high morbidity and mortality, causing a large burden with respect to health expenditure [1,2]. Patients with HF have poor prognoses, with a re-hospitalization rate of >50% and mortality rate of 13–31% within 1 year depending on the severity of HF [1,3,4,5]. 

Natriuretic peptides such as brain natriuretic peptide (BNP) and N-terminal pro-BNP (NT-proBNP) are secreted by the heart in response to hemodynamic change as well as neuro-hormone and immune systems disturbances [6], which play a key role in the regulation of cardiovascular and renal function [7]. These natriuretic peptides are synthesized and stored by atrial and ventricular cardiomyocytes as prohormones (for example proBNP) which are subsequently split into two fragments including the biologically inactive NT-proBNP and biologically active BNP at the time of secretion to the bloodstream. Besides the above-mentioned “classical” scenario, the processing of human proBNP to active BNP was also demonstrated to occur in the circulation [8]. The stimulators of production and/or secretion of BNP by cardiomyocytes include not only the dilatation of atrial and ventricular cardiomyocytes, but also some hormones and drugs such as endotelin-1, glucocorticoids, thyroid hormones, and alfa-adrenergic agonists. [7] The biological actions of BNP include natriuresis, diuresis, and vasodilation, as well as the direct suppression of volume-retaining and vasoconstricting systems [9]. Because of the longer plasma half-life and lower intra-individual biological variation, NT-proBNP is considered a more reliable biomarker than BNP in clinical practice. Clinically, the plasma NT-proBNP level grossly reflects cardiac structure and function in HF patients [10,11,12], has good diagnostic performance for discriminating acute HF from a noncardiac entity in patients with new-onset dyspnea, and has good prognostic–predictive power in HF patients [13,14,15,16,17]. Noveanu et al. [17] conducted a study to compare the prognostic–predictive power of individual NT-proBNP level obtained at different time points. In this prospective multi-center study enrolling 171 patients with acute decompensated HF presenting at the emergency department, plasma NT-proBNP levels were obtained at presentation, 24 h, 48 h, and pre-discharge states. The predictive powers of NT-proBNP levels for one-year mortality increased stepwise as the hospitalization course continued (the area under the curve (AUC) of the receiver operating characteristic (ROC) curve values were 0.67, 0.73, 0.75, and 0.77 at presentation, 24 h, 48 h, and pre-discharge states, respectively). Only the pre-discharge NT-proBNP level could independently predict one-year mortality [17].

The plasma NT-proBNP levels would change following therapies [18,19,20,21], and it is reasonable to consider that the latest measurement of NT-proBNP has the most potent predictivity to subsequent outcomes. It is also reasonable to doubt that a single measurement of a biomarker for which levels vary along with the treatment course could predict long-term patient outcomes. Previously, several investigations demonstrated the prognostic–predictive power of the levels of NT-proBNP by using various study designs including the type of HF, patient ages, and follow-up periods of endpoints [13,14,15,16,17]. Nonetheless, none of these studies compared the predictivity of plasma NT-proBNP level for mortality with different follow-up periods among the same participant setting in the single study.

We hypothesized that in patients with hospitalization-requiring acute HF, the association between plasma NT-proBNP levels at initial presentation and all-cause mortality would decrease along with the increased follow-up period, and conducted the current study to test this hypothesis.

## 2. Experimental Section

### 2.1. Study Design and Population

This retrospective study was conducted using a previously-built acute HF cohort [22] which was established in a regional teaching hospital in Taiwan from January 1, 2011, to December 31, 2013.

The inclusion criteria of the current study included adult hospitalized patients with HF (International Classification of Diseases-9, codes 428, 428.0, 428.1, and 428.9) as the final diagnosis at discharge, confirmed by: (1) the age-related cut-points of plasma NT-proBNP levels which were drawn within 24 h of hospitalization [13]; and (2) echocardiography during hospitalization. The exclusion criteria were patients aged below 18 years, patients with severe chronic pulmonary diseases (i.e., forced expiratory volume in one second <1 liter in pulmonary function), decompensated hepatic diseases with ascites, and renal failure necessitating renal replacement therapy. For patients who were hospitalized for more than once, only the first hospitalization was counted in the current study. The participant selection and data collection have been detailed in our previous work [22].

Information obtained from patients’ medical charts included baseline demographic data, comorbid diseases, Charlson comorbidity index (CCI), etiologies of HF, and the New York Heart Association Functional Classification (NYHA Fc), along with other clinical parameters including medications, vital signs and laboratory results at initial presentation, echocardiography reports, chest roentgenogram, and electrocardiogram. In the patients admitted to intensive care unit (ICU), additional information including the use of a mechanical ventilator, noninvasive positive pressure ventilation (NIPPV), or inotrope support, the experience of cardiopulmonary resuscitation (CPR), the length of stay (LOS) in the hospital and ICU, and in-hospital mortality as the outcome parameter were also documented.

### 2.2. NT-proBNP Assay

The NT-proBNP values were measured using the non-competitive electrochemiluminescence automated immunoassay with two monoclonal antibodies (Roche Diagnostics GmbH, Mannheim, Germany) [23]. This assay is the most used method for the measurement of NT-proBNP, with excellent diagnostic accuracy and clinical relevance [24].

### 2.3. Endpoints of the Study

The primary endpoint of the current study was 12-month mortality. The censoring period was defined from the initial hospitalization to mortality (in non-survivors) or on the 365th day (in survivors). The secondary endpoint included 1-month, 3-month, 6-month, and 9-month mortality. The endpoints of participants were obtained by chart reviews and telephone visits. 

### 2.4. Ethical Approval

The current study was reviewed and approved by the Institutional Review Board of Saint Mary’s Hospital Luodong (No. SMHIRB-103004), and was carried out by the approved guidelines. Informed consents were waived because of no breach of privacy and interference with clinical decisions.

### 2.5. Statistical Analysis

We performed the statistical analyses using the Scientific Package for Social Science (PASW Statistics for Windows, Version 23.0, SPSS Inc, Chicago, IL, USA) and R 3.3.2 (R Foundation for Statistical Computing, Vienna, Austria) software. Kurtosis and skewness tests were used as normal distribution tests for continuous variables. Categorical variables were reported as case number (percentage). Normal distribution continuous variables were expressed as mean ± standard deviation, and non-normal continuous distribution variables were additionally provided median (range) values. The independent *t*-test and chi-squared test were applied to compare continuous and categorical variables, respectively, between two groups. For non-normal distribution variables, the statistical comparisons were performed after log transformation of their values.

The NT-proBNP levels were transformed into categorical variables in advance by using the best cut-point which was determined by the generalized additive model (GAM), for measuring the mortality risk. The Kaplan–Meier curve with the log-rank test was used to compare the survival probability between groups. The Cox proportional hazard regression model with the enter and forward stepwise methods were applied to determine the risk factors of mortality in univariate and multivariate fashions, respectively. 

The variables which were significantly different between 12-month survivors and non-survivors were put into the multivariate analysis to find the independent predictors of 12-month mortality. The variables which were significantly correlated with mortality at the corresponding follow-up periods were taken as candidate variables for determining risk factors of 1-month, 3-month, 6-month, and 9-month mortalities. The elimination criterion for the multivariate analysis was set at *p* >0.05. In all statistical analyses, a two-sided *p* <0.05 was taken as statistical significance.

## 3. Results

During the study period, in total 1276 patients were screened, and 1007 patients were excluded (990 patients due to lack of final diagnosis of HF at discharge, age younger than 18 years, severe chronic pulmonary disease, decompensated hepatic disease with ascites, or renal failure requiring renal replacement therapy, and 17 patients owing to lack of echocardiography examinations). Finally, a total of 269 patients (mean age, 74.5 ± 13.6 years; female, 53.9 %) were enrolled in the current study and 72 patients died within the 12-months follow-up period. The causes of death included 26 (36.1% among non-survivors) due to cardiac reasons, with 8 (11.1%) experiencing sudden cardiac arrest, and 46 (63.9%) with non-cardiac reasons. According to the primary endpoint, 12-month mortality, all patients were categorized as survivors (*n* = 197, 73.2 %) or non-survivors (*n* = 72, 26.8 %).

### 3.1. Basic Characteristics, Clinical Variables, and Outcomes

Comparing to the 12-month survivors, the non-survivors were older (80.9 ± 10.6 versus 72.1 ± 13.9) and had higher CCI (8.8 ± 2.6 versus 6.5 ± 2.5), but there were fewer smokers (15.3% versus 27.9%), and a lower body mass index (22.5 ± 4.6 versus 24.0 ± 5.3). The non-survivors had higher proportion of chronic kidney disease (65.3% versus 35.5%), malignancy (9.7% versus 3.6%), and higher NYHA Fc. They were less likely to take cardiovascular agents such as angiotensin-converting enzyme inhibitor (ACEI)/angiotensin receptor blocker (ARB) (37.5% versus 60.4%), beta-blocker (19.4% versus 41.6%), aldosterone blocker (16.7% versus 31.5%), and loop diuretics (48.6% versus 65.5%). At initial presentation, the non-survivors had lower hemoglobin (10.6 ± 2.4 versus 11.9 ± 2.4 g/dL) and estimated glomerular filtration rate (eGFR) (44.9 ± 35.5 versus 57.8 ± 33.2 mL/min/1.73 m^2^), but a higher neutrophil percentage (78.5 ± 13.7 versus 72.7 ± 12.7%) and NT-proBNP (14,966.8 ± 12,724.6 (median, 10,116.5) versus 10,275.2 ± 11,591.6 (median, 5977.0) ng/L). Regarding the reports of echocardiography, the percentage having “left ventricular hypertrophy” was significantly higher in the non-survivors (51.4% versus 36.0%), whereas the percentages with left ventricular ejection fraction (LVEF), different types of heart failure (HF with preserved ejection fraction (HFpEF)/HF with midrange ejection fraction (HFmrEF)/HF with reduced ejection fraction (HFrEF)), dilated left atrium, and dilated left ventricle were not statistically different between survivors and non-survivors. During hospitalization, the non-survivors also had a higher proportion of infection (70.8% versus 44.2%), and were more likely to receive mechanical ventilation (29.2% versus 14.2%), noninvasive positive pressure ventilation (NIPPV) (19.4% versus 7.1%), vasopressors (25.0% versus 7.6%), and CPR (12.5% versus 2.0%). They also had a longer LOS in the ICU (5.08 ± 7.53 versus 2.56 ± 4.58 days). All *p* <0.05 (Table 1).

### 3.2. Association of Plasma NT-pro-BNP Levels and 12-Month Mortality

**Figure 1:**
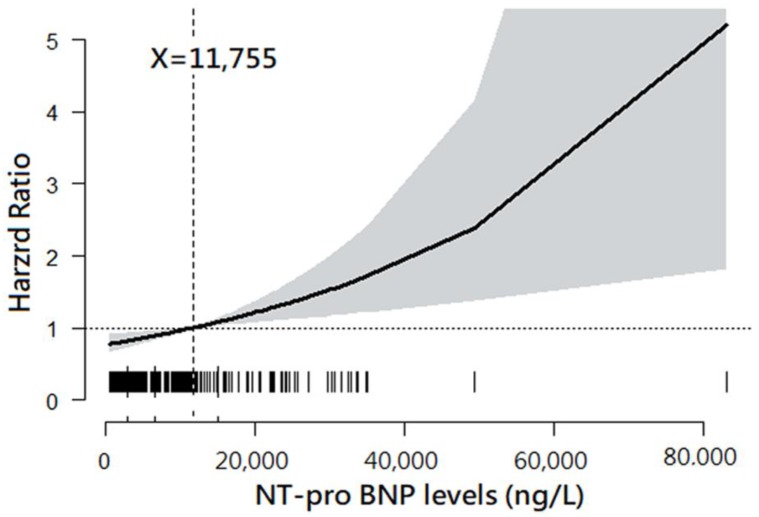
Association between plasma N-terminal pro-brain natriuretic peptide (NT-proBNP) levels and 12-month mortality. Note: The plot was drawn using a general additive model. The best cut-point of plasma NT-proBNP level was 11,755 ng/L.

The plot drawn by GAM demonstrated a trend of increasing HR for 12-month mortality along with increased NT-proBNP levels. Moreover, the NT-proBNP level of 11,755 ng/L was found to be the best cut-off point at which the HR of 12-month mortality was 1.0 (Figure 1).

**Figure 2:**
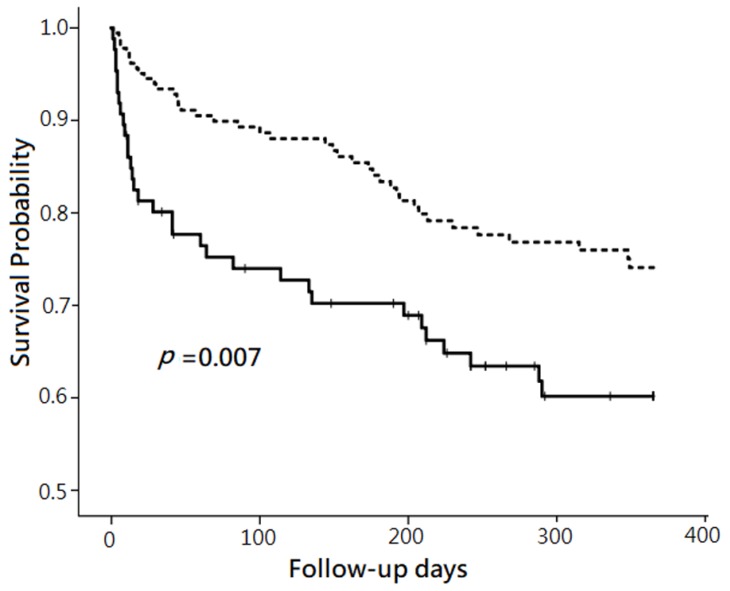
Comparison of 12-month mortality between two groups categorized by plasma N-terminal pro-brain natriuretic peptide (NT-proBNP) levels. Note: The comparison was performed without risk factor adjustment using the Kaplan–Meier method. The patients with plasma NT-proBNP ≧11,755 ng/L (solid line) had significantly lower survival probability than those with plasma NT-proBNP <11,755 ng/L (dashed line) after follow-up for 12 months (*p* = 0.007).

By using univariate Cox proportional hazard regression analysis, the hazard ratio (HR) of 12-month mortality was 1.88 for the patients with NT-proBNP level ≧11,755 ng/L, compared to those with NT-proBNP level <11,755 ng/L. The plot made by Kaplan–Meier method showed that the survival probability after follow-up for 12 months was lower in the patients with plasma NT pro BNP level ≧11,755 ng/L than those with plasma NT pro BNP level <11,755 ng/L (*p* = 0.007). (Figure 2) When setting the HR as 1.88 and α as 0.05, the calculated power of the Cox proportional hazard regression using R software in the current study was 0.92.

### 3.3. The Independent Predictors of 12-Month Mortality

After putting the variables with statistical differences in the comparison between survivors and non-survivors into a multivariate Cox proportional hazard regression model, we found five independent predictors. Higher “CCI” (adjusted HR (aHR) = 1.22; 95% confidence interval (CI), 1.10–1.34), increased “age” (aHR = 1.07; 95% CI, 1.04–1.10), “administration of vasopressor” (aHR = 3.43; 95% CI, 1.76–6.71), and “underwent CPR” (aHR = 4.59; 95% CI, 1.86–11.31) positively predicted 12-month mortality, but “with ACEI/ARB” (aHR = 0.41; 95% CI, 0.25–0.68) provided survival benefits during 12-month follow-up (all *p* <0.001) (Table 2). Nevertheless, “plasma NT-proBNP level ≧11,755 ng/L” (aHR = 1.41; 95% CI, 0.88–2.26; *p* = 0.138) did not show significant predictive power in predicting 12-month mortality in the multivariate analysis. 

### 3.4. Predictive Powers of Plasma NT-proBNP Levels on Mortality with Various Follow-Up Periods

**Figure 3:**
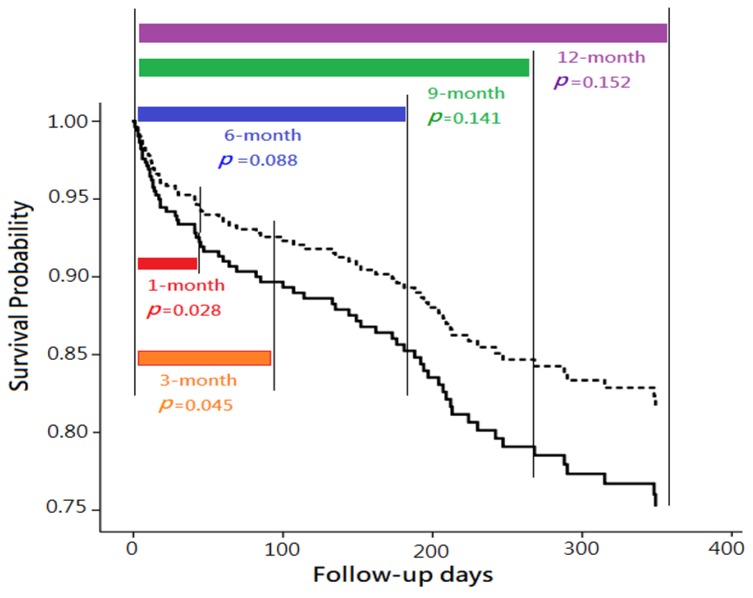
Comparison of mortality with various follow-up periods, between two groups categorized by plasma N-terminal pro-brain natriuretic peptide (NT-proBNP) levels, with risk factor adjustment. Note: The comparisons were performed using multivariate Cox proportional hazard regression models. The survival differences of between the patients with plasma NT-proBNP ≧11,755 ng/L (solid line) and those with plasma NT-proBNP <11,755 ng/L (dashed line) were only observed in 1-month (*p* = 0.028) and 3-month follow-up (*p* = 0.045), but not found in longer follow-up (including 6-month, 9-month, and 12-month follow-up).

Subsequently, Cox proportional hazard regression model was applied to evaluate the predictive power of plasma NT-proBNP level on 1-month, 3-month, 6-month, 9-month, and 12-month mortalities. In univariate analysis, “plasma NT-proBNP level ≧11,755 ng/L” was significantly associated with higher mortality within the follow-up periods of 1-month, 3-months, 6-months, 9-months, and 12-months (all *p* <0.01). However, in multivariate analyses,“plasma NT-proBNP level ≧11,755 ng/L” was only found as an independent predictor of mortality in 1-month (aHR = 2.37; 95% CI, 1.10–5.11; *p* = 0.028) and 3-month follow-up (aHR = 1.98; 95% CI, 1.02–3.86; *p* = 0.045), but not found in more extended follow-up (including 6-month, 9-month, and 12-month follow-up) (Table 3 and Figure 3). 

**Figure 4:**
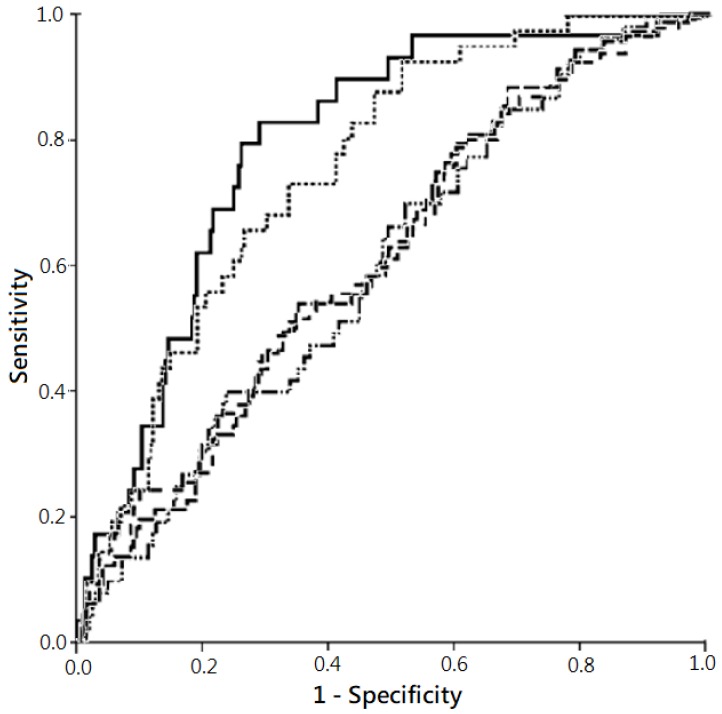
Predictivities for all-cause mortality by plasma N-terminal pro-brain natriuretic peptide (NT-proBNP) levels in varied follow-up periods, with risk factor adjustment. Note: The comparison was performed using receiver operating characteristic (ROC) curve. The plot showed the highest predictability for all-cause mortality in 1-month follow-up (solid line; area under the curve (AUC) of ROC (AUC ROC), 0.79; 95% confidence interval (CI), 0.71–0.87; *p* <0.001)), followed subsequently by 3-month follow-up (dotted line; AUC ROC, 0.76; 95% CI, 0.69–0.83; *p* <0.001), 9-month follow-up (dash-dotted line; AUC ROC, 0.62; 95% CI, 0.54–0.69; *p* = 0.005), 12-month follow-up (dashed line; AUC ROC, 0.62; 95% CI, 0.54–0.69; *p* = 0.004), and 6-month follow-up (dash-solid line; AUC ROC, 0.61; 95% CI, 0.53–0.69; *p* = 0.016).

Besides, the AUC ROC denoting the predictive power for all-cause mortality by “plasma NT-proBNP level >11,755 ng/L” was higher in 1-month (0.79) and 3-month mortality (0.76), but relatively lower in more extended follow-up periods (Figure 4).

## 4. Discussion

### 4.1. NT-proBNP and Patient Prognoses

Since the discovery of the relationship of NT-proBNP and cardiac function [25], an increasing body of evidence has proved the usefulness of this biomarker for diagnosis, risk stratification, and monitoring in HF patients [26,27]. In the current study, we disclosed the reverse association between the predictivity of plasma NT-proBNP levels for all-cause mortality and follow-up period among acute HF patients. 

Using the outpatient-based HF population enrolled at emergency departments, both Gegenhuber et al. [28] and Andersson et al. [16] demonstrated that higher plasma NT-proBNP level in a single measurement was associated with higher 1-year mortality. The 1-year mortality rate significantly elevated to 2.6-fold when the plasma NP-pro BNP above 2060 ng/L [28], or increased to 7.0-fold with the plasma NP-pro BNP above 10,000 ng/L [16]. Another outpatient-based investigation by Sokhanvar et al. [14], which enrolled patients with systolic HF, also disclosed that the level of plasma NT-proBNP was positively correlated with mortality and morbidity at a follow-up period of six months. Besides, the study by Gardner et al. [15] found that, for advanced chronic HF patients, a single level of plasma NT-proBNP >1505 ng/L (the median level of the participants) independently predicted of all-cause mortality within 554 days.

Contrary to the above-mentioned studies in which the single plasma NT-proBNP level could predict morbidity and mortality in a more extended follow-up period (six months or longer) in relatively stable or chronic HF patients, Januzzi et al. [13] found that a higher presenting NT-proBNP (>5180 ng/L) could only predict mortality in a shorter follow-up period (76 days) in acute HF patients. These results were in line with our findings that, in severe acute HF patients requiring hospitalization, higher plasma NT-proBNP levels at initiation presentation could only independently predict shorter-term (i.e., one month and three months), but not longer-term mortality (more than three months). 

There are several potential explanations for this finding. First, patients with acute decompensated HF are of the extremely high risk of mortality in the first few months of hospitalization [29]. Second, the plasma NT-proBNP levels of admitted HF patients would significantly decline with clinical improvements following short-term therapy [18,19]. Furthermore, changes in NT-pro BNP during follow-up are associated with parallel changes in morbidity and mortality in acute HF [30]. Thus, serial NT-proBNP measurements are recommended as a powerful predictor of clinical outcomes [30,31]. Third, the factors affecting plasma NT-proBNP levels, such as age, renal impairment, pulmonary problems, vascular problems, and malignancies [9] also played a role as independent predictors of mortality in the current study. The influence of NT-proBNP on mortality might be masked.

### 4.2. Other Predictors of Mortality

The five independent predictors of 12-month mortality included “age”, “CCI”, “with ACEI/ARB”, “administration of vasopressor”, and “underwent CPR.” In the current study, the risks increased 7% and 22% with increases of 1 year of age and 1 point of CCI, respectively. It is well known that age and comorbidities affect prognoses of patients in many settings. Specifically, advanced age was found to be associated with increased short and long-term mortality in hospitalized patients with HF [32], whereas CCI, the most widely used comorbidity index, was strongly correlated with patients outcomes in hospitalized HF patients [33]. As to ACEI/ARB, these two kinds of agents were suggested for improving symptoms and decreasing mortality risk in HF patients [34]. ACEI was demonstrated to lower the risk of mortality in patients with HF [31]. Moreover, ARBs are recommended in patients with symptomatic HF who are ACEI intolerant to reduce mortality [34]. 

In the current study, “underwent CPR” and “administration of vasopressor” were independently associated with 4.59- and 3.43-fold increased 12-month mortality rates, respectively, compared to those without the two individual types of management. “Experience of CPR” was disclosed to carry poor in-hospital survival and long-term survival following discharge in a retrospective study enrolling 732 patients experiencing CPR for in-hospital cardiac arrest [35]. In addition, “administration of vasopressors” was associated with in-hospital CPR and identified as an independent predictor of in–hospital mortality in a multicenter registry including 4153 hospitalized acute HF patients [2]. Clinically, the need for CPR and vasopressors is indicative of an exhaustion of compensatory mechanisms to maintain hemodynamics and circulation. It is not surprising to know that these “exhausted” patients have unfavorable prognoses. Besides, the adverse effects of “using vasopressors” [36] might additionally increase the mortality risk.

It is worthy of discussion that, in the current study, the “LVEF” at initial presentation did not show the outcome-predictive power, and the 12-month mortality risk was similar in patients with LVEF≧50% (HFpEF) compared to either those with LVEF <50% (HFmrEF+HFrEF) (29.5% versus 23.8%, *p* = 0.296) or even those with LVEF <40% (HFrEF) (29.5% versus 25.3%, *p* = 0.485). Since the enrolled participants were hospitalized patients with acute HF, the LVEF data obtained during the hospitalization might reflect acutely deteriorated heart function rather than chronic stable status. Thus it is reasonable that LVEF as well as the types of HF defined by the LVEF were of poor predictive power for patient outcomes in the current study.

Besides LVEF, echocardiographic global longitudinal strain (GLS), a simple parameter expressing the percentage of left ventricular (LV) longitudinal shortening, is also a potential examination. It was not only suggested as a practical tool for surveillance of LV systolic dysfunction [37], but also demonstrated as a more predictive parameter than LVEF for 5-year mortality in the patient with acute HF [38]. Further investigation is encouraged to evaluate the associations among NT-proBNP levels, GLS, and prognoses of HF patients. 

Recently, a multicenter randomized controlled trial enrolling 881 patients from 129 sites confirmed the safety and efficacy of angiotensin receptor–nephrilysin inhibitors (ARNIs) in hospitalized patients with acute decompensated HF [39]. Compared with the patients taking enalapril, the patients randomized to ARNI experienced a significantly greater decrease in NT-proBNP level during the therapy, along with a significantly reduced risk of composite adverse clinical outcomes including death, rehospitalization for HF, implantation of a left ventricular device, and inclusion on a heart transplant eligibility list [39]. These findings linked the associated between NT-proBNP levels and patient prognoses, and also recommend ARNI as a good tool for further investigation in the cardiology field.

### 4.3. Limitations

Several limitations of the current study exist. First, this was a retrospective study which was subject to bias. Second, the investigation focused on the relationship between clinical prognosis and a single plasma NT-proBNP measurement at the initial presentation. The serial changes in plasma NT-proBNP levels after management during hospitalization were not available. Third, the current study was conducted using a relatively high severity acute HF cohort in which about half of participants were admitted to ICU. Thus the findings may not be extendedly applied to chronic HF patients. Fourth, the enrolled participants number (*n* = 269) might be not high enough to increase the number of subgroups for multivariate regression analyses. Lastly, there was no external validation by another independent cohort. Further multi-center, prospective investigations are warranted to confirm the predictive power in varied follow-up periods and to explore the relationship between the serial measurement of plasma NT-proBNP and patient outcomes. 

## 5. Conclusions

In the current study, a single measurement of plasma NT-proBNP could predict mortality within three months of follow-up, but not the mortality of longer follow-up in hospitalized acute HF patients. The authors demonstrated a reverse association between the predictivity of plasma NT-proBNP levels for all-cause mortality and a longer follow-up period in this population. Alternatively, serial measurements of plasma NT-proBNP might be needed for monitoring patient responses to treatment or clinical status [20]. 

## Figures and Tables

**Table 1:** Comparisons of basic characteristics and clinical variables between survivors and non-survivors after a 12-month follow-up.

	Survivor (*n* = 197)	Non-Survivor (*n* = 72)	*p*-Value
Age, years	72.1 ± 13.9 74.0 (75.0)	80.9 ± 10.6 83.0 (55.0)	<0.001
Gender, female	104 (52.8%)	41 (56.9%)	0.545
Current smoker	55 (27.9%)	11 (15.3%)	0.033
Body mass index, kg/m^2^	24.0 ± 5.3 23.4 (27.0)	22.5 ± 4.6 22.1 (28.3)	0.006
**Charlson comorbidity index**	6.5 ± 2.5	8.8 ± 2.6	<0.001
**Comorbidity**			
Chronic lung disease	44 (22.3%)	13 (18.1%)	0.447
Diabetes mellitus	80 (40.6%)	35 (48.6%)	0.240
Chronic kidney disease	70 (35.5%)	47 (65.3%)	<0.001
Cerebral vascular accident	21 (10.7%)	13 (18.1%)	0.106
Malignancy	7 (3.6%)	7 (9.7%)	0.044
**Heart-specific comorbidity**			0.776
Hypertension	12 (6.1%)	4 (5.6%)	
Ischemic heart disease	90 (45.7%)	36 (50.0%)	
Rheumatic heart disease	4 (2.0%)	2 (2.8%)	
Valvular heart disease	74 (37.6%)	27 (37.5%)	
Dilated cardiomyopathy	17 (8.6%)	3 (4.2%)	
**Medication**			
ACEI/ARB	119 (60.4%)	27 (37.5%)	0.001
Beta-blocker	82 (41.6%)	14 (19.4%)	0.001
Ald. blocker	62 (31.5%)	12 (16.7%)	0.016
Loop-diuretic	129 (65.5%)	35 (48.6%)	0.012
Digoxin	34 (17.3%)	6 (8.3%)	0.069
**Vital sign at initial hospitalization**			
Body temperature, °C	36. 5 ± 0.8 36.3 (5.8)	36.4 ± 0.8 36.3 (4.4)	0.736
Heart rate, bpm	95.7 ± 23.4 95.0 (120.0)	97.7 ± 24.8 98.3 (100.0)	0.474
Respiratory rate, breath per min	22.9 ± 5.3 21.0 (39.0)	22.9 ± 5.6 22.0 (30.0)	0.843
Systolic blood pressure, mmHg	138.9 ± 31.1 137.0 (172.0)	138.5 ± 32.3 138.0 (148.0)	0.592
Diastolic blood pressure, mmHg	82.9 ± 18.4 82.0 (111.0)	76.5 ± 15.6 78.0 (76.0)	0.187
**NYHA Fc**			<0.001
II	53 (26.9%)	15 (20.8%)	
III	110 (55.8%)	28 (38.9%)	
IV	34 (17.3%)	29 (40.3%)	
**Laboratory tests at initial hospitalization**			
White blood cell, ×10^9^/L	9.9 ± 4.4 8.7 (24.8)	10.6 ± 6.1 9.2 (36.3)	0.762
Hemoglobin, g/dL	11.9 ± 2.4	10.6 ± 2.4	<0.001
Neutrophil, %	72.7 ± 12.07 73.8 (60.9)	78.5 ± 13.7 80.3 (64.7)	0.009
Sodium, mmol/L	138.4 ± 5.6 139.0 (39.0)	136.1 ± 6.6 137.0 (30.0)	0.007
Potassium, mEq/L	4.0 ± 0.8 3.9 (5.1)	4.2 ± 1.0 4.0 (4.5)	0.374
eGFR, ml/min/1.73m^2^	57.8 ± 33.2 54.9 (169.9)	44.9 ± 35.5 37.6 (216.0)	0.002
Glutamate oxaloacetate transaminase, U/>	55.0 ± 119.9 29.0 (1349.0)	35.7 ± 26.0 28.0 (126.0)	0.603
Sugar (non-fasting), mg/dL	179.3 ± 114.7 146.0 (1005.0)	174.3 ± 94.3 148.2 (678.0)	0.819
NT-proBNP, ng/L	10,275.2 ± 11,591.6 5,977.0 (82,590.0)	14,966.8 ± 12,724.6 10,116.5 (48,195.0)	0.001
White blood cell, ×10^9^/L	9.9 ± 4.4 8.7 (24.8)	10.6 ± 6.1 9.2 (36.3)	0.762
Hemoglobin, g/dL	11.9 ± 2.4	10.6 ± 2.4	<0.001
Electrocardiogram-AF	60 (30.5%)	27 (37.5%)	0.274
LVEF, %	48.2 ± 16.8 49.0 (78.0)	50.8 ± 17.6 51.5 (73.0)	0.391
Types of HF			0.494
HFpEF (LVEF ≧50%)	98 (49.7%)	41(56.9%)	
HFmrEF (LVEF 40-49%)	31 (15.7%)	8 (11.1%)	
HFrEF (LVEF <40%)	68 (34.5%)	23 (31.9%)	
Left ventricular hypertrophy	71 (36.0%)	37 (51.4%)	0.023
Dilated left atrium	118 (59.9%)	36 (50%)	0.146
Dilated left ventricle	78 (39.6%)	22 (30.6%)	0.174
Pulmonary hypertension	59 (29.9%)	21 (29.2%)	0.901
Infection during hospitalization	87 (44.2%)	51 (70.8%)	<0.001
**Outcomes**			
ICU admission	95 (48.2%)	44 (61.1%)	0.061
Length of ICU stay, days	2.56 ± 4.58	5.08 ± 7.53	0.001
Mechanical ventilator	28 (14.2%)	21 (29.2%)	0.005
Length of ventilator, days	10.27 ± 10.33	9.29 ± 13.01	0.779
Use of NIPPV	14 (7.1%)	14 (19.4%)	0.003
Length of NIPPV, days	4.21 ± 4.21	4.86 ± 4.90	0.713
Administration of vasopressor	15 (7.6%)	18 (25.0%)	<0.001
Underwent CPR	4 (2.0%)	9 (12.5%)	<0.001

Note: Data are expressed as *n* (%), mean ± standard deviation or median (range). The independent *t*-test and chi-squared test were used to compare continuous and categorical variables, respectively, between survivors and non-survivors. Abbreviations: ACEI = angiotensin converting enzyme inhibitors, AF = atrial fibrillation, Ald. blocker = aldosterone receptor blocker, ARB = angiotensin receptor blocker, CPR = cardiopulmonary resuscitation, eGFR = estimated glomerular filtration rate, HF = heart failure, HFmrEF = HF with midrange ejection fraction, HFpEF = HF with preserved ejection fraction, HFrEF = HF with reduced ejection fraction, ICU = intensive care unit, LVEF = left ventricular ejection fraction, NIPPV = noninvasive positive pressure ventilator, NYHA Fc = New York Heart Association Functional Classification, NT-proBNP = N-terminal pro-brain natriuretic peptide.

**Table 2:** Independent predictors of 12-month mortality.

	B	aHR (95% CI)	*p*-Value
**Age**	0.07	1.07 (1.04–1.10)	<0.001
**Charlson comorbidity index**	0.20	1.22 (1.10–1.34)	<0.001
**With ACEI/ARB**			
**No**	Reference		
**Yes**	−0.89	0.411 (0.25–0.68)	<0.001
**Administration of vasopressor**			
**No**	Reference		
**Yes**	1.23	3.43 (1.76–6.71)	<0.001
**Underwent CPR**			
**No**	Reference		
**Yes**	1.52	4.59 (1.86–11.31)	<0.001

**Note:** The analysis was performed by using multivariate Cox proportional hazard regression model. The predictors which exhibited significant differences between survivors and non-survivors at 12-month follow-up (shown in Table 1) were put into the multivariate Cox proportional hazard regression for analysis, with an elimination criterion of *p* >0.05, to investigate their regression coefficient, adjusted hazard ratio, and *p*-values. Abbreviations: ACEI = angiotensin-converting enzyme inhibitors, aHR = adjusted hazard ratio, ARB = angiotensin receptor blocker, B = Cox regression coefficient, CI = confidence interval, CPR = cardiopulmonary resuscitation.

**Table 3:** Comparisons of independent predictors for mortality with various follow-up periods.

	1-month Mortality ^1^	3-month Mortality ^2^	6-month Mortality ^3^	9-month Mortality ^4^	12-month Mortality ^5^
**Crude HR (95% CI)**					
PlasmaNT-pro BNP level (≧ 11,755 ng/L)	3.32 (1.59–6.59) **	2.76 (1.49–5.09) **	2.12 (1.24–3.64) **	1.90 (1.18–3.08) **	1.88 (1.18–2.99) **
**aHR (95% CI)**					
					
Plasma NT-proBNP level (≧11,755 ng/L)	2.37 (1.10–5.11) *	1.98 (1.02–3.86) *	1.63 (0.93–2.86)	1.44 (0.89–2/35)	1.41 (0.88–2.26)
Age	1.05 (1.01–1.10) *	1.06 (1.03–1.10) ***	1.05 (1.02–1.09) **	1.07 (1.04–1.10) ***	1.07 (1.04–1.10) ***
Administration of vasopressor	3.59 (1.34–9.67) *	5.35 (2.30–12.43) ***	3.65 (1.75–7.61) **	374 (1906–7.39) ***	3.43 (1.76–6.71) ***
Underwent CPR	5.93 (1.99–17.64) **	4.04 (1.31–12.47) *	5.62 (2.16–14.64) ***	4.70 (1.90–11.65) **	4.59 (1.86–11.31) ***
With ACEI/ARB	0.23 (0.08–0.61) **	0.27 (0.13–0.58) **	0.42 (0.23–0.76) **	0.43 (0.26–0.73) ***	0.411 (0.25–0.68) ***
With loop-diuretic	0.37 (0.15–0.90) *	—	—	—	—
With beta-blocker	—	0.13 (0.30–0.53) **	0.33 (0.15–0.75) **	—	—
With ICU admission	—	0.36 (0.14–0.90) *	—	—	—
With mechanical ventilator	—	3.46 (1.19–10.11) *	—	—	—
With CPAP	—	3.70 (1.43–9.63) **	—	—	—
Charlson comorbidity index	—	—	1.18 (1.05–1.32) **	1.21 (1.09–1.34) ***	1.22 (1.10–1.34) ***

Note: The analyses were performed using multivariate Cox proportional hazard regression. The variables put into the multivariate analyses included those which were significantly correlated with mortality at 1-month, 3-month, 6-month, 9-month, and 12-month follow-up. ^1^ In predicting 1-month mortality, the “plasma NT-proBNP level” was adjusted to “age”, “administration of vasopressor”, “underwent CPR”, “with loop-diuretic”, and “with ACEI/ARB.” ^2^ In predicting 3-month mortality, the “plasma NT-proBNP level” was adjusted to “age”, “with ICU admission”, “with NIPPV”, “with a mechanical ventilator”, “administration of vasopressor”, “underwent CPR”, “with beta-blocker”, and “with ACEI/ARB”. ^3^ In predicting 6-month mortality, the “plasma NT-proBNP level” was adjusted to “age”, “administration of vasopressor”, “underwent CPR”, “with beta-blocker”, and “with ACEI/ARB”. ^4^ In predicting 9-month mortality, the “plasma NT-proBNP level” was adjusted to “age”, “administration of vasopressor”, “underwent CPR”, “Charlson comorbidity index”, and “with ACEI/ARB.” ^5^ In predicting 12-month mortality, the “plasma NT-proBNP level” was adjusted to “age”, “administration of vasopressor”, “underwent CPR”, “Charlson comorbidity index”, and “with ACEI/ARB.” *, **, and *** denote *p* <0.05, <0.01, and <0.001, respectively. — denotes that the variable was not put into the multivariate analyses because of being excluded in the correlation analyses. Abbreviations: ACEI = angiotensin-converting enzyme inhibitor, aHR = adjusted hazard ratio, ARB = angiotensin receptor blocker, CI = confidence interval, CPAP = continuous positive airway pressure, CPR = cardiopulmonary resuscitation, HR = hazard ratio, ICU = intensive care unit, NT-proBNP = N-terminal-pro-B-type natriuretic peptide.
